# Draft genome sequence of the basidiomycetous yeast *Rhodosporidiobolus colostri* DBVPG 10655, a cold-adapted degrader of lignin-derived monoaromatic compounds

**DOI:** 10.1128/mra.01105-25

**Published:** 2026-04-20

**Authors:** Caroline Poyntner, Benedetta Turchetti, Pietro Buzzini, Rosa Margesin

**Affiliations:** 1 Department of Microbiology, University of Innsbruck 27255, Innsbruck, Austria; 2 Department of Agricultural, Food and Environmental Sciences, Industrial Yeasts Collection DBVPG, University of Perugia 9309, Perugia, Italy; University of California Riverside 8790, Riverside, California, USA

**Keywords:** cold-adapted, yeast, forest soil, biodegradation

## Abstract

Here, we report the draft genome sequence of *Rhodosporidiobolus colostri* DBVPG 10655. The cold-adapted basidiomycetous yeast was isolated from Alpine forest soil and can degrade lignin-derived monoaromatic compounds.

## ANNOUNCEMENT

Yeasts belonging to the genus *Rhodosporidiobolus* (phylum Basidiomycota) have been isolated from various ecological niches, including cold ecosystems, marine ecosystems, and waste deposit (1, 2). *R. colostri* DBVPG 10655 was isolated at 4°C from soil from an Alpine deciduous forest site in South Tyrol, Italy (46°25′36.8″ N, 11°17′48.6″ E) (3). The strain is able to degrade lignin-derived monoaromatic compounds (p-coumaric acid, 4-hydroxybenzoic acid, ferulic acid, and vanillic acid) over a broad temperature range (5–25°C) (4). The strain was previously identified using PCR amplification of the internal transcribed spacer region (ITS1 and ITS2) using standard primers (ITS1 and ITS2). ITS sequences were queried against the NCBI database using BLAST (5).

For the full genome sequencing, the strain was grown from a single colony on R2A agar and was further inoculated in R2A broth incubated at 10°C until the stationary growth phase. For DNA extraction, the innuPrep DNA Mini Kit (innu screen GmbH) was used, adding Lyticase for cell wall digestion. After quality and quantity testing using Nanodrop (Thermo Fisher Scientific) and Quantus (Promega), the DNA was randomly sheared and used for library preparation using the Novogene NGS DNA Library Prep Set (Cat no. PT004) following the manufacturer’s recommendations. The DNA was sequenced using Illumina Novaseq 6000 PE150 at Novogene Sequencing, resulting in 4,785 Mbp raw data with 150 bp long reads. For all software tools, default parameters were used. Quality control and filtering of raw reads were performed with Fastp (v0.20.0) (6) removing low-quality bases (mass value ≤ 20), the number of *N* in reads beyond 10 bp and reads overlapping more than 15 bp with the adapters. The clean data comprised 3,988 Mbp with 58.81% GC content. For the assembly, SPAdes (7) software was used followed by contig integration with CISA (8) integration (Table 1).

**TABLE 1:** Assembly statistics and results of the functional annotation from each database

Assembly	
Genome size	24.5 Mb
Contigs total number	1,844
Contigs *N*_50_	26.7 kb
Contigs Max length	135,224 bp
Contigs Min length	501 bp
Coverage	158×
**Database (Reference, kind of prediction**)	**Number of predicted coding genes**
SwissProt (9, proteins)	1,899
KEGG (10, pathways)	3,442
COG (11, eukaryotic Proteins)	1,617
TCDB (12, transport proteins)	373
GO (13, functions)	4,341
Pfam (14, protein families)	4,341
PHI (15, pathogen-host interactions)	666

For homology-based gene prediction, protein sequences from other phylogenetically relative species were aligned to the genome using BLAST (5), followed by GeneWise (16) and coding gene prediction using Augustus (version 2.7) (17). This resulted in 1.4 × 10^7^ predicted genes. The functional annotation (Table 1) showed genes linked to xenobiotic-related pathways, which supports previous biodegradation results (4).

The strain is a good candidate for further research on degradation processes, especially in cold environments.

## Data Availability

The sequences for strain identification can be found in the DBVPG database using strain number 10655 (https://dsa3.unipg.it/DBVPG/en/lievito/?id=10655). The draft genome sequence of *Rhodosporidiobolus colostri* DBVPG 10655 has been deposited in the GenBank database under JBRIID000000000, the raw data in NCBI SRA under SRR35824689, and the functional annotation in FigShare under 10.6084/m9.figshare.31220656.
